# Pericytes Derived from Adipose-Derived Stem Cells Protect against Retinal Vasculopathy

**DOI:** 10.1371/journal.pone.0065691

**Published:** 2013-05-31

**Authors:** Thomas A. Mendel, Erin B. D. Clabough, David S. Kao, Tatiana N. Demidova-Rice, Jennifer T. Durham, Brendan C. Zotter, Scott A. Seaman, Stephen M. Cronk, Elizabeth P. Rakoczy, Adam J. Katz, Ira M. Herman, Shayn M. Peirce, Paul A. Yates

**Affiliations:** 1 Department of Ophthalmology, University of Virginia, Charlottesville, Virginia, United States of America; 2 Department of Pathology, University of Virginia, Charlottesville, Virginia, United States of America; 3 Department of Molecular Physiology and Pharmacology, Tufts University, Boston, Massachusetts, United States of America; 4 Center for Ophthalmology and Visual Sciences, Department of Molecular Ophthalmology, The University of Western Australia, Nedlands, Western Australia, Australia; 5 Department of Plastic Surgery, University of Virginia, Charlottesville, Virginia, United States of America; 6 Department of Biomedical Engineering, University of Virginia, Charlottesville, Virginia, United States of America; Cedars-Sinai Medical Center, United States of America

## Abstract

**Background:**

Retinal vasculopathies, including diabetic retinopathy (DR), threaten the vision of over 100 million people. Retinal pericytes are critical for microvascular control, supporting retinal endothelial cells via direct contact and paracrine mechanisms. With pericyte death or loss, endothelial dysfunction ensues, resulting in hypoxic insult, pathologic angiogenesis, and ultimately blindness. Adipose-derived stem cells (ASCs) differentiate into pericytes, suggesting they may be useful as a protective and regenerative cellular therapy for retinal vascular disease. In this study, we examine the ability of ASCs to differentiate into pericytes that can stabilize retinal vessels in multiple pre-clinical models of retinal vasculopathy.

**Methodology/Principal Findings:**

We found that ASCs express pericyte-specific markers *in vitro*. When injected intravitreally into the murine eye subjected to oxygen-induced retinopathy (OIR), ASCs were capable of migrating to and integrating with the retinal vasculature. Integrated ASCs maintained marker expression and pericyte-like morphology *in vivo* for at least 2 months. ASCs injected after OIR vessel destabilization and ablation enhanced vessel regrowth (16% reduction in avascular area). ASCs injected intravitreally before OIR vessel destabilization prevented retinal capillary dropout (53% reduction). Treatment of ASCs with transforming growth factor beta (TGF-β1) enhanced hASC pericyte function, in a manner similar to native retinal pericytes, with increased marker expression of smooth muscle actin, cellular contractility, endothelial stabilization, and microvascular protection in OIR. Finally, injected ASCs prevented capillary loss in the diabetic retinopathic Akimba mouse (79% reduction 2 months after injection).

**Conclusions/Significance:**

ASC-derived pericytes can integrate with retinal vasculature, adopting both pericyte morphology and marker expression, and provide functional vascular protection in multiple murine models of retinal vasculopathy. The pericyte phenotype demonstrated by ASCs is enhanced with TGF-β1 treatment, as seen with native retinal pericytes. ASCs may represent an innovative cellular therapy for protection against and repair of DR and other retinal vascular diseases.

## Introduction

Aberrant retinal angiogenesis and vasculopathies, including retinopathy of prematurity (ROP), exudative age-related macular degeneration (AMD), and DR, are among the leading causes of irreversible vision loss throughout the world [1]. DR alone afflicts an estimated 101 million people worldwide, with a prevalence of 155 million expected by 2030 [2]. While the pathogenesis and timing that underlie each condition differ, each involves destabilization of the retinal microvasculature.

Retinal pericytes, the cells that ensheath the retinal microvasculature, play a key role in the stabilization of endothelial cells, protecting them from hypoxic insults and angiogenic stimuli [3]. They are likely to be amongst the first cellular responders to diabetes–induced complications, a direct consequence of chronic diabetic inflammation [4], [5]. Inadequate pericyte coverage of microvasculature has also been implicated in the pathogenesis of ROP [6], [7]. Rich in actin [8], contractile pericytes are considered critical for microvascular control throughout the human body, supporting endothelial cells via contact-dependent [9] and soluble mediator-driven mechanisms [10], [11]. Following pericyte death or loss, endothelial dysfunction results in downstream hypoxic insult, increased vascular endothelial growth factor (VEGF) production, macular edema, pathologic angiogenesis, and ultimately blindness [12].

Previous work has elegantly demonstrated that intravitreally-injected bone marrow-derived stem cells (BMSCs) can help ameliorate and repair retinopathic insults. However, BMSCs appear to act primarily through their incorporation into the retina as endothelial cells, microglia, and photoreceptors [13], [14], [15], [16], [17]. Although pericytes can be derived from BMSCs [18], this does not appear to be a predominant differentiation pathway for these cells when injected into the eye [14], [19].

ASCs are an alternative type of adult mesenchymal stem cells that are readily isolated from subcutaneous fat and contain a vascular progenitor subpopulation that is easily expanded for use in regenerative medicine [20]. Importantly, these cells are postulated to have a direct role in providing microvascular support and appear to differentiate readily as pericytes [20], [21]. These attributes of ASCs suggest they could be useful for treatment of retinal microvascular disease.

To examine their potential therapeutic application in retinal vasculopathy, we sought to determine whether ASCs, once injected intravitreally, are able to migrate to and integrate with the retinal microvasculature. We also assessed the extent to which ASCs could replicate characteristics of endogenous retinal pericytes in *in vitro* assays of retinal pericyte function. We hypothesized that, as pericyte progenitors, ASCs could enable microvascular stabilization and offer protection against retinal vascular insults, including diabetic retinopathy.

## Materials and Methods

### Cell Harvest

Human adipose derived stem cells **(**hASCs) were obtained from patients under full approval of University of Virginia’s Institutional Review Board. Adipose tissue was removed surgically from patients during elective plastic surgery by Adam Katz, MD, in the operating room at the University of Virginia, as previously described [22]. To prepare hASCs for *in vitro* and *in vivo* assays, the stromal vascular fraction was isolated from patient-derived lipoaspirate and further purified with serial passaging and sub-culturing, as we and others have previously shown [22]. Human bone marrow derived stem cells (hBMSCs) were purchased from Allcells (#MSC-001F). Bovine retinae and retinal microvascular endothelial cells (BRECs) and pericytes were isolated and characterized as previously described [8], [23], [24], [25]. The tissues were received from an abattoir and no institutional animal use committee approval was required. Mouse adipose derived stem cells (mASCs) were purchased from Eton Bioscience (280004001G) for functional studies or isolated from the epididymal fat pads of 9-week old Akita mice for integration studies [26], [27]. The epididymal fat pads were digested in collagenase-containing digestion buffer (0.01% collagenase type I, 2.5% BSA, 200 nM adenosine, 20 mM HEPES, 120 mM NaCl, 4.7 mM KCl, 1.3 mM CaCl_2_-2H_2_O, 1.2 mM KH_2_PO_4_, 1.2 mM MgSO_4_-7H_2_O) for one hour at 37C. The resulting mixture was filtered through 200-µμ mesh to exclude any undigested tissue. The filtrate was centrifuged to remove remaining collagenase, and the pellet (containing cells) incubated with red blood cell lysis buffer (15.5 mM NH_4_Cl, 10 mM KHCO_3_, 0.1 mM EDTA) for 5 minutes at room temperature to lyse red blood cells. The cell suspension was then sterile-filtered through 40-µμ mesh and plated on sterile culture plates.

### Cell Culture

Isolated hASCs and mASCs were cultured in 10 cm Nunc culture dishes (Thermoscientific #12-565-020) using Gibco DMEM/F12 (1∶1) (Life Technologies #11320-033), supplemented with 10% Gibco fetal bovine serum (Invitrogen #16000044, Lot #953873), 1% antibiotic-antimycotic (Invitrogen #15240062). hBMSCs were cultured in the same media as hASCs, but augmented to 20% fetal bovine serum. Media was replaced every three days and cells were passaged at 70% confluency using Stempro Accutase (Gibco/Life Technologies #A11105-01). Cells were maintained at 37°C, 5% CO_2_, and 75% humidity. hASCs and mASCs were used from passage 3 to 6 in all studies. hBMSCs were used at passage 3. Bovine retinae and retinal microvascular endothelial cells and pericytes were isolated and characterized as previously described [8], [25].

### Cellular Contractility Assay

The assay was performed essentially as described [28]. Briefly, round 12 mm diameter cover slips were coated with a thin layer of silicone that was then thermally cross-linked. Coverslips were charged using a glow discharge apparatus and were coated with Type I collagen suspended in PBS (0.1 mg/mL, BD Biosciences #354236). The prepared cover slips were placed in the wells of 24-well tissue culture plates, sterilized for 3 minutes with UV radiation, and seeded with 2×10^3^ hASCs or bovine retinal pericytes. Cellular contraction caused deformation of the silicone substrate, which was visualized by brightfield imaging. 24 hours after plating, hASCs were either left in serum-containing media or treated with media containing 10 ng/mL recombinant human TGF-β1 (rhTGF-β1, R&D Systems #240B). Following another 24 hour incubation period, control or rhTGF-β1-treated hASCs were visualized with an Axiovert 200 M microscope (Carl Zeiss MicroImaging, Thornwood, NY) using 5× or 10× objective lenses [25], [28]. In order to quantify changes in hASC contractility induced by rhTGF-β1, we determined the percentage of wrinkling cells cultured in the presence or absence of rhTGFβ-1. The values were recorded using Microsoft Excel and are shown as a mean +/− SD.

### Co-Culture Assay

This assay was performed as described previously [25] with the following modifications. UV-sterilized round cover slips were placed into the wells of 24-well tissue culture plates and seeded with either bovine retinal pericytes or hASCs at a density of 3×10^3^ cells/well. After the cells adhered overnight, cultures were either incubated with normal 10% serum-containing growth media or pre-conditioned with 1 ng/mL rhTGF-β1 in 0.5%–5% serum-containing media for 24–48 hours. Pericyte and hASC cultures were washed with PBS to remove the rhTGFβ-1, and BRECs were added to the wells at a density of 5×10^3^ cells/well in 5% serum-containing media. For control experiments, BRECs were plated in monoculture, and allowed to attach overnight. For BREC monoculture, half the wells were treated with 5% serum media while the remainder was treated with 1 ng/mL rhTGF-β1 in 5% serum-containing media. Both mono- and co-cultures were incubated for 24 hours, then S-phase entry (endothelial proliferation) was assessed using the Click-iT® EdU Cell Proliferation Kit (Invitrogen #C10339) according to the manufacturer’s instructions. Following EdU detection, hASC and pericyte cultures were stained using mouse anti-smooth muscle actin (SMA) (Biogenex #MU128-UC) and anti-mouse Alexa-Fluor 488 secondary antibody (Invitrogen A-21200). Fluorescence and phase contrast imaging was performed following fixation and staining as previously described [25]. Endothelial cell S-phase entry in control and treated co-cultures both for BRECs that were isolated or those that were contacting either hASCs or bovine retinal pericytes was then quantified and values expressed +/− SD.

### *In Vitro* Immunohistochemistry

hASCs and mASCs were briefly washed in PBS, fixed using methanol (10 min at −20°C), rinsed with PBS (3×5 min), blocked with 1% BSA, 2% goat serum, 0.1% Triton X in PBS, exposed to primary antibodies in blocking solution (4°C overnight), washed with PBS (3×10 min), and either cover slipped or exposed to secondary antibody (45 min), followed by washing with PBS (3×10 min). Antibodies used for hASCs were mouse monoclonal SMA-FITC (Sigma-Aldrich F3777; 1∶250), rabbit polyclonal NG2 (Millipore AB5320; 1∶100), rabbit polyclonal PDGFRβ (Santa Cruz sc-432; 1∶100), and goat anti rabbit Cy2 secondary (Millipore; 1∶250). mASCs were stained with anti-SMA preconjugated to Cy3 (Sigma #C6198-.2ML).

### Flow Cytometry

Antibody characterization of hASCs (passage 4) was performed on a Becton Dickinson/Cytek FACSCalibur C with CellQuest Pro acquisition software, using FlowJo software for analysis. Cells were lifted using Accutase (20 min), fixed with methanol (10 min at −20°C), washed with 1% BSA in PBS, and permeabilized with 0.1% Triton X, 1% BSA in PBS (10 min at 4°C). Primary antibodies were incubated in 1% BSA in PBS (45 min), washed with 1% BSA in PBS (2×10 min), and re-suspended in 500 µL 1% BSA in PBS for analysis. Antibodies used for hASCs were mouse monoclonal β-actin-FITC (Abcam ab64496), mouse monoclonal SMA-FITC (Sigma-Aldrich F3022; 1∶500), rabbit polyclonal PDGFRβ (Santa Cruz sc-432; 1∶200), mouse IgG1-FITC isotype control (BD Pharmingen 554679; 0.5 ug/uL), and goat anti rabbit Cy2 secondary (Millipore; 1∶250). Results were replicated using at least 3 separate experiments. mASCs (passage 4) were evaluated on a BD FacsCanto II with 405 nm, 488 nm and 633 nm lasers. mASCs were stained with PDGFRβ (CD140b) preconjugated to APC (eBioscience #17-1402-80, 1∶200), α-smooth muscle actin preconjugated to Cy3 (Sigma #C6198-.2ML, 1∶200).

### Ocular Injections

Mice were anesthetized with ketamine/xylazine injected intraperitoneally or with inhaled isoflurane. If palpebral fissures were not yet open, they were carefully cut with iris scissors, followed by local application of proparacaine. Prior to injection, hASCs, mASCs, or hBMSCs were stained with 1,′-Dioctadecyl-3,3,3′,3′-tetramethylindocarbocyanine perchlorate (DiI, Invitrogen D282), using a standard labeling technique for tracking of adult mesenchymal stem cells [29], [30], [31], [32], [33], [34]. hASCs, mASCs, or hBMSCs were suspended in 0.5 to 1.5 uL of PBS and were injected into the vitreous of the study eye with a 33 gauge Hamilton needle through the pars plana. An equal volume of PBS was injected into the contralateral eye to serve as a paired control. For palpebral fissures cut with iris scissors, a small amount of methylcyanoacrylate was used to re-seal the lids. All injected ASCs were passage 4 to passage 6 at the time of injection. BMSCs were passage 3 at the time of injection.

### Oxygen Induced Retinopathy (OIR)

All procedures performed with mice conformed to the guidelines within the ARVO Statement for the Use of Animals in Ophthalmology and Vision Research and were approved by the University of Virginia’s Animal Care and Use Committee. NOD SCID mice (Charles River) were immersed in 94% oxygen from postnatal day 7 (P7) to day 12 (Biospherix A30274-P, P-110-E702), as 75% oxygen did not produce substantial central avascular area in NOD SCID pups. The mice were checked through the clear plastic door twice daily for the duration of the oxygen treatment. Retinas were harvested as described below and retinal wholemounts prepared. Areas of capillary pruning (major vessels remain intact) were traced manually by blinded individuals and quantified in ImageJ. Following OIR, 10,000 hASCs in 1.5 µL of PBS were injected into the vitreous of the P12 eye, with an equal volume of PBS alone injected into the contralateral control eye. As a cell type control for the OIR recovery model, two litters (n = 10 P12 pups) of NOD SCID mice were used with either 10,000 hBMSCs or 10,000 hASCs in 1.5 µL of PBS injected into the vitreous of the P12 eye, and 1.5 µL of PBS alone in the contralateral control eye. To account for litter variability, half the mice in these litters received hBMSCs (n = 5 eyes of 5 P12 pups) in the study eye, while the other half received hASCs (n = 5 eyes of 5 P12 pups) in the study eye. Use of two NOD SCID litters controlled for known litter and littermate variability. All eyes were harvested and examined for areas of capillary pruning at P14. For the OIR protection model, 600 hASCs suspended in 0.5 µL of PBS were injected at P2 into the study eye and 0.5 µL of PBS alone in the contralateral control eye. Eyes were harvested and examined for areas of capillary pruning at P12 following OIR. For the rhTGF-β1 OIR protection model, 1000 hASCs were suspended in 0.5 µL of PBS after 48 hours of rhTGF-β1 pretreatment and subsequent media replacement. They were then injected at P6 into one eye and 1000 untreated hASCs suspended in 0.5 µL of PBS were injected into in the contralateral eye. Eyes were harvested and examined for areas of capillary pruning at P12 following OIR.

### Akimba Model of Diabetic Retinopathy

Kimba and Akita mice were obtained from Elizabeth Rakoczy, PhD, and crossed as previously described to establish a breeding colony for further ASC studies [35]. For functional microvascular studies, intraocular injections for the Akimba vascular protection model were performed at P9 with mASCs (Eton Bioscience, passage 3) cultured as described (*Cell Harvest, Ocular Injections*). Specifically 1,000 mASCs in 0.5 µL of PBS were injected into the vitreous of the P9 eye, with equal volume of PBS alone in the contralateral eye. Two months after injection, fluorescein angiography was performed by intraperitoneal injection of 0.1 mL of 10% fluorescein reconstituted in PBS into mice anesthetized with Ketamine/Xylazine. A Cantor Nissel contact lens was applied to the cornea and the retinal vasculature imaged on a Heidelberg Spectralis retinal imager as previously described. Following angiography, retinas were harvested and retinal wholemounts prepared as described below in the *Retinal Wholemounts* section of Methods. Areas of capillary dropout, as defined by areas that have lost tertiary retinal branches of retinal microvasculature, were circumscribed by blinded observers and recorded in ImageJ. In a second experiment we examined the potential for homologous incorporation of mASCs at late time points. For this latter experiment, mASCs were harvested from Akimba mouse epididymus, cultured, and then subsequently injected (as described above in *Cell Harvest* section of Methods). Specifically 10,000 mASCs in 1.5 µL of PBS were injected into the vitreous of the 5 week old Akimba eye (n = 8 eyes of 8 mice), with equal volume of PBS alone in the contralateral eye. Retinas were harvested one month later at 9 weeks of age and wholemounts prepared (as described below in the *Retinal Wholemounts* section of Methods). Retinal vessels were lectin and SMA stained, and imaged for incorporation of DiI-labeled mASCs into the retina and association with the retinal microvasculature.

### Retinal Wholemounts

Mice were sacrificed by overdose of ketamine/xylazine injected intraperitoneally and then perfused for 5 minutes with 4% paraformaldehyde by insertion of a needle into the left ventricle of the heart, attached to a perfusion pump, following cutting of the inferior vena cava. Eyes were then enucleated and immersed in 4% paraformaldehyde for 10 minutes, without any perforation of eye, before being transferred to PBS at room temperature. Cornea, extraocular muscles, optic nerve, iris, sclera, retinal pigment epithelium, lens, and hyaloid vasculature were then carefully removed in that order. Four relaxing cuts were made to enable flatmounting on a gelatin-coated slide. After permeabilization with 1 mg/mL digitonin (MP Biomedicals 0215948050) for 1 hour, retinas were stained with mouse monoclonal SMA-FITC (Sigma-Aldrich F3022; 1∶250), isolectin IB4-647 (Invitrogen I32450; 1∶200), isolectin IB4-488 (Invitrogen I21411; 1∶200), anti-NG2 chondroitin sulfate proteoglycan (Millipore AB5320; 1∶100), and goat anti-rabbit IgG Cy2-conjugated (Millipore AP132J; 1∶250). Slides were mounted using fluorogel with TRIS buffer (Electron Microscopy Sciences 17985-10).

### Data Quantification and Statistics

Retinas were imaged using a Leica TCS SP2 confocal with DMIRE2 inverted microscope and a Zeiss LSM 510 confocal microscope. 10× images were montaged using i2kAlignRetina (Dual Align, LLC) or the MosaicJ plugin in the Fiji Software version of ImageJ. Figures were constructed in Adobe Photoshop CS5. Except where indicated, in order to be conservative with statements of statistical significance in light of non-Gaussian dissimilar variance, comparisons were made using median values and significance tested with nonparametric Wilcoxan Matched Pairs and Mann-Whitney tests, calculated on GraphPad Prism (version 6.00 for Macintosh, GraphPad Software, La Jolla California USA, www.graphpad.com). Error bars represent standard deviation. Statistical significance is denoted in all figures in the manuscript by asterisks with * for p≤0.05, and ** for ≤0.01, and *** for ≤0.001.

## Results and Discussion

### Retinal Incorporation of Intravitreally Injected hASCs

We initially sought to determine if ASCs could be efficiently delivered to the retina through intravitreal pars plana injection, and once there, whether they would successfully integrate with the retinal vasculature. We injected hASCs intravitreally, similar to previously published BMSC retinal incorporation experiments [13]. In particular, our goal was to assess the ability of intravitreally delivered ASCs to assume abluminal locations around retinal capillaries, which is a defining characteristic of pericytes.

In the OIR model, the central retinal microvasculature is ablated during a hyperoxic insult that takes place from P7 to P12 [12], while the major vessels remain. Revascularization of the central retina, including recruitment of native retinal pericytes, occurs once mice are returned to room air at P12 [36]. hASCs suitable for injection were derived from human lipoaspirates that were serially passaged in culture, due to the known enrichment of a pericyte-like phenotype at later passages [37]. Before injection, hASCs were labeled with DiI, as used by others for tracking adult mesenchymal stem cells *in vivo* [29], [30], [31], [32], [33], [34]. Initial injections with 10,000 DiI-labeled hASCs suspended in sterile PBS were performed through the pars plana at P12 after mice had first been exposed to OIR [14], [38], [39], [40], [41].

**Figure 1:**
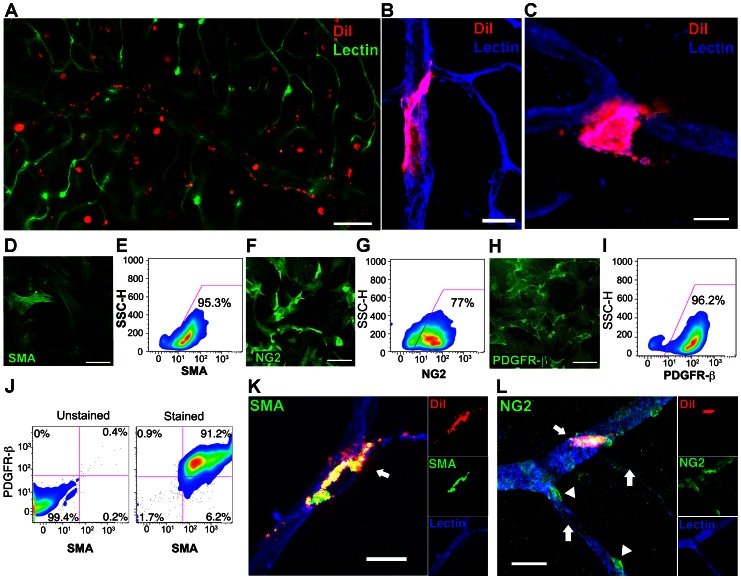
hASCs demonstrate pericyte-like morphology and phenotype markers *in vitro* and *in vivo*. **A** **B–C**, DiI-labeled hASCs (red) wrap around isolectin labeled retinal microvessels (blue) abluminally and target vascular junctions, both properties of terminally differentiated pericytes. **D–I**, Passage 5 hASCs exhibit *in virto* expression of the characteristic pericyte markers smooth muscle actin (SMA, **D–E**), nerve/glial antigen 2 (NG2, **F–G**), and platelet derived growth factor receptor beta (PDGFR-β, **H–I**) by both immumohistochemical staining on cultured cells and by flow cytometry on cells harvested from these cultures. **J**, 91.2% of hASCs demonstrated colabeling of SMA and PDGFR-β compared to unstained controls. **K–L**, DiI labeled hASCs injected intravitreally into NOD SCID mice at P12, after OIR hyperoxia, maintain intimate association with the retinal microvasculature 6–8 weeks later and demonstrate persistent SMA (**K**), NG2 (**L**) (white arrows), with SMA extending into the cellular extension wrapping around the capillary. On average 308 hASCs, or 3.08% of the 10,000 injected cells, remained engrafted, with 85.6% of engrafted hASCs found in physical contact with retinal microvessels. Note native retinal pericytes are also labeled with NG2 (**L**) but lack DiI staining (white arrowheads). In contrast, no native retinal pericytes are seen labeled with SMA (**K**) given that this is a tertiary branch of the retinal vasculature, and native pericyte SMA expression is typically seen only on primary and secondary vessels. Scale bars: **A** = 200 µm, **B = **20 µm, **C** = 10 µm, **D, F, H** = 100 µm, **K** = 10 µm, **L** = 20 µm.

hASCs were observed in abundance alongside central retinal microvasculature 10 days after injection (Fig. 1A). 8 weeks after injection, 308 hASCs, or 3.08% (2.08–4.07% 95% CI) of the 10,000 injected cells, remained engrafted, which is comparable to stem cell engraftment efficiencies observed in other model systems [42]. 85.6% (82.8–88.7% 95% CI) of engrafted hASCs were found in physical contact with retinal microvessels, many adopting pericyte-like abluminal locations and phenotypic wrapping around vessels (Fig. 1B, C). While our studies to date do not conclusively rule out some proliferation of injected ASCs after injection into the eye, the numbers of cells observed two months after injection were not qualitatively more than the number of cells in eyes harvested one week after injection.

### Verification of hASC-derived Pericyte Marker Expression *in vitro* and *in vivo*

While a functional pericyte phenotype for hASCs is suggested by their morphology and perivascular location, we sought to explore hASC expression of characteristic pericyte markers. Alpha smooth muscle actin (SMA), nerve/glial antigen 2 (NG2), and platelet derived growth factor receptor beta (PDGFRβ) frequently designate a pericyte phenotype, although specific isoprotein expression is dependent upon microvascular context *in vivo* [43], [44]. We found that hASCs express abundant steady state protein levels characteristic of native microvascular pericytes, including SMA, NG2, and PDGFRβ as measured by both antibody staining and flow cytometry (Fig. 1D–I). 91.2% of hASCs tested co-expressed both SMA and PDGFRβ (Fig. 1J). Additionally, hASCs injected intravitreally into NOD SCID mice at P12, following OIR induction, also continue to express SMA and NG2, when harvested six to eight weeks post-injection (Fig. 1K–L). Both our *in vivo* and *in vitro* studies indicate that serially passaged hASCs can differentiate into retinal pericytes in response to microenvironmental cues.

Importantly, no ocular tumor formation was observed and no metastatic lesions in the lung or liver were found at these time points, consistent with prior reports on *in vivo* hASC use [45]. Sagittal sections of hASC injected eyes stained with either Oil Red O or Alcian Blue did not reveal any off target differentiation of hASCs into adipose or chondrocyte lineages (data not shown). Similarly, antibody staining with F4/80, a microglial and macrophage marker, did not show any definitive differentiation into these cell types. Interestingly, we saw no significant cell trafficking to deeper layers of the retina on sagittal sections or 3D confocal reconstruction of the retinal vasculature. Sub-retinal hASCs were sometimes seen near the site of injection, often associated with a localized retinal detachment, suggesting that trafficking to these locations occurred at the time of injection.

### hASCs Accelerate Recovery from Oxygen Induced Retinopathy

**Figure 2:**
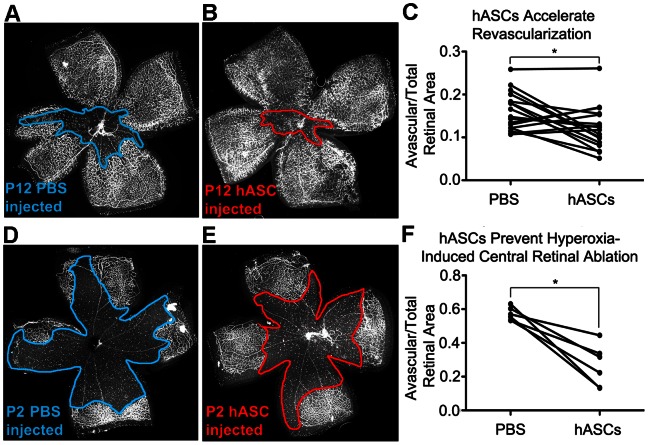
Human adipose-derived stem cells (hASCs) stabilize oxygen-induced retinopathy (OIR). **A–C**, Eyes of NOD SCID mice, injected intravitreally with hASCs at P12 following OIR, and then harvested at P14, demonstrate improved revascularization of central retina as compared to contralateral PBS injected (blue) carrier controls (16.4% reduction, n = 17, p = 0.03). **D–F**, NOD SCID eyes injected intravitreally with hASCs at P2, prior to OIR, exhibited dramatically less central vascular ablation at P12 than contralateral PBS injected carrier controls (blue) (52.9% reduction, n = 6, p = 0.03). Lines in **C** and **F** connect data points for hASC injected and contralateral PBS injected eyes in the same mouse.

Having demonstrated that hASCs could home to and integrate with retinal vasculature with stable expression of pericyte markers, we next assessed whether these injected hASCs would functionally affect revascularization of the central retina following OIR injury. As before, eyes were injected with hASCs at P12 following OIR and then harvested at P14, which in NOD-SCID mice is the midpoint of post-OIR revascularization. Contralateral eyes were injected with an equal volume of PBS vehicle, serving as a commonly employed carrier control [14], [46], [47], [48], [49], and allowing paired comparison of eyes. NOD-SCID mice were chosen to avoid the potential immune rejection of our xenografted hASCs that might confound our analysis of revascularization. Flat-mounted retina demonstrated a 16.4% reduction in avascular area (n = 17, p = 0.03), indicating greater central retinal revascularization compared to contralateral control retinas injected with PBS (Fig. 2A–C). No appreciable pre-retinal neovascularization was observed at any time point in either control or hASC injected eyes, likely due to our use of immunocomprimised mice, since inflammatory stimuli have been shown to be a large component of the aberrant neovascular response [50].

We performed an additional experiment to benchmark hASCs against a cell-type control. Two NOD SCID litters (n = 10 pups total) were injected with either 10,000 hASCs (n = 5 eyes of 5 pups) or 10,000 human bone marrow derived stem cells (hBMSCs) (n = 5 eyes of 5 pups) in the study eye at P12, along with an equal volume of PBS in the contralateral control eye (n = 10 eyes of 10 pups). Unsorted hBMSCs were chosen as a negative control cell since sorted lineage negative murine BMSCs are another mesenchymal stem cell previously shown to influence retinal vasculature [13]. Consistent with the earlier results of this study, hASCs accelerate revascularization of the central retina by 16.4% at P14 compared to contralateral PBS injected control eyes (n = 5 pups, p = 0.03). However, hBMSCs had no statistically significant effect on the rate of revascularization compared to contralateral PBS injected control eyes (n = 5 pups, p = 0.29) (Fig. S1).

### hASCs Protect against Oxygen Induced Retinopathy

We next determined whether injected hASCs could protect central retinal microvessels against destabilization and ablation seen during OIR. Eyes were injected at P2 with 600 DiI labeled hASCs suspended in 0.5 µl PBS due to the smaller ocular volume at P2, with contralateral eyes receiving equal volumes of PBS as a vehicle control. Notably, injection of hASCs at P2 does not appear to appreciably affect normal retinal vascularization through P7 (Fig. S2). Injected mice underwent OIR from P7 to P12 per standard protocol [36] but with oxygen concentration elevated to 94% required for NOD SCID central retinal microvascular ablation. Retinal wholemounts at P12 demonstrated a profound 52.9% reduction of avascular area (n = 6, p = 0.03) in hASC-treated eyes as compared to contralateral PBS-injected controls (Fig. 2D–F).

This result suggests that injected hASCs may assist in stabilizing retinal microvasculature that is otherwise acutely unstable. Although the mechanism underlying this protection is not yet clear, we have found that injected hASCs efficiently integrate into the retinal microvasculature after P8 and we observe a 57% increase (n = 16 and 12, p = 0.031) in vascular length density in retinal sub-fields containing labeled cells as compared to sub-fields without cells. This finding is consistent with prior studies demonstrating decreased vessel susceptibility to OIR corresponding to an increased desmin ensheathment ratio, which serves as a surrogate for pericyte coverage [6], [7].

However, this explanation seems insufficient to account fully for the observed results. Few cells are seen integrated with the retinal microvasculature at P7, prior to OIR, with less than 5% of total injected cells found associated with the microvasculature by P12, following OIR. Given that ASCs are known to secrete a large repertoire of trophic factors and that the majority of injected cells remain in the vitreous upon dissection, its seems likely that hASCs mediate protection of retinal microvessels at least in part via paracrine signaling mechanisms.

### hASCs Respond to Pre-treatment with TGF-β1 in a Manner Analogous to Endogenous Retinal Pericytes

**Figure 3:**
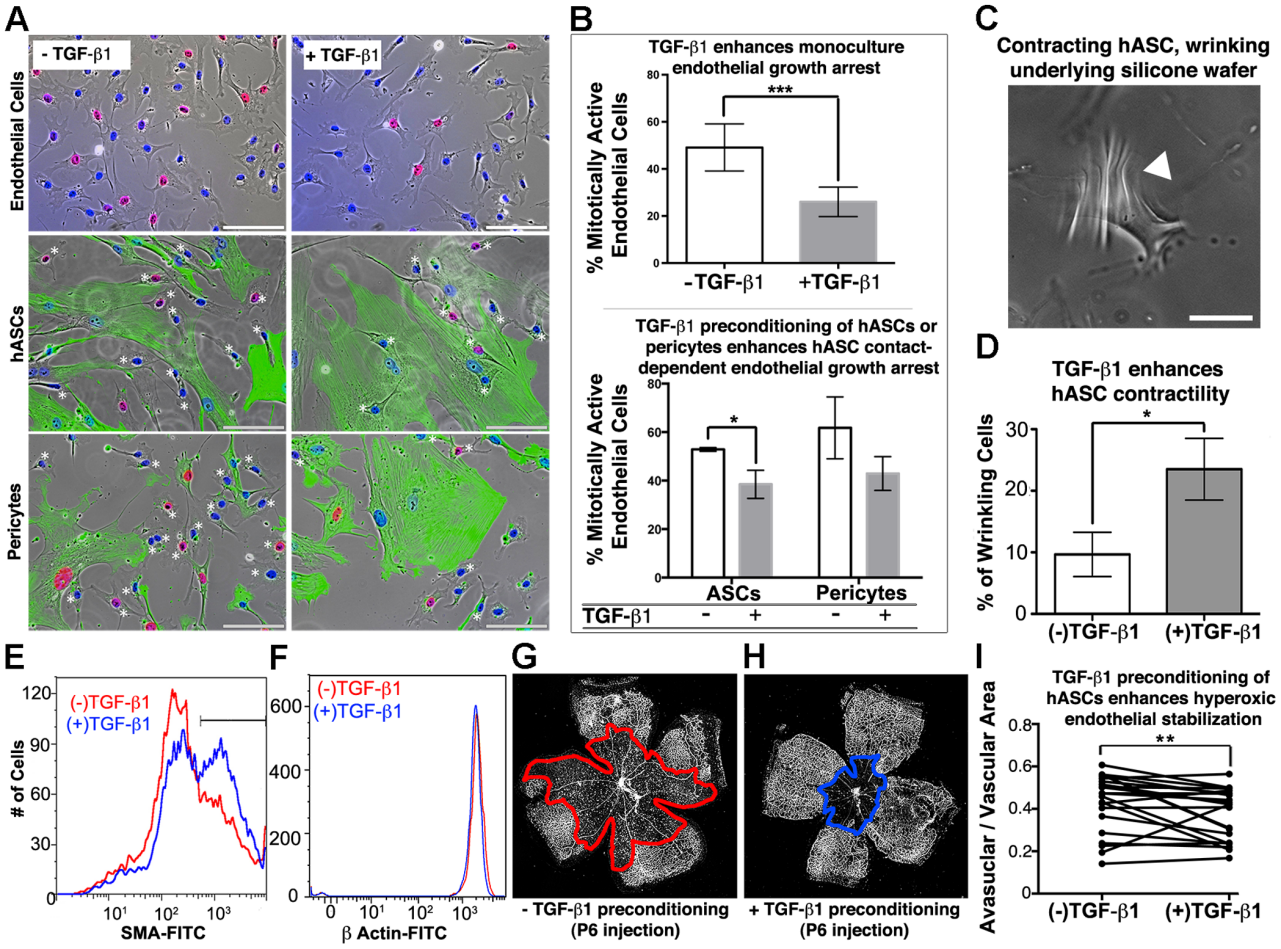
hASC-pericytes respond to TGF-β1 treatment in a manner analogous to native retinal pericytes. **A**, **B**, TGF-β1 τρεατμεντ of endothelial monoculture and pre-conditioning of hASC/pericyte co-cultures (right panels) are compared to control serum conditions (left panels). Endothelial cells demonstrate a 23.1% decrease in S-phase nuclei, or mitotic entry (pink nuclei), when treated with TGF-β1 alone (p<0.01). Further, endothelial cells in direct contact (asterisks) with hASCs and pericytes (labeled green with SMA antibodies) demonstrate a 14.4% and 15.7% decrease, respectively, in cell cycle entry when hASC/pericytes are pre-treated with TGF-β1 (π<0.05). **C**, **D**, TGF-β1 πρε−τρεατμεντ significantly strengthens pericyte-like contractile phenotype as measured by hASC ability to deform underlying silicone substrates (white arrowhead) by 2.6 fold (n = 32 treated cells and 40 untreated cells, p = 0.0413), a response analogous to that seen with bovine retinal pericytes [53] **.** **E**, **F**, 48-hour pre-treatment of cultured hASCs with TGF-β1 increases the percentage of hASCs highly expressing SMA by 15.3%, without altering β actin expression. **G**–**I**, Eyes intravitreally injected with TGF-β1 treated hASCs at P6, demonstrated an 11.0% (n = 22, p = 0.01) reduction in retinal avascular area upon removal from hyperoxia at P12, as compared to contralateral eyes injected with untreated hASCs. Scale bars: **A**, **C** = 100 µm.

TGF-β1 has been previously demonstrated to have a direct effect on retinal pericytes and influence their potential interactions with endothelial cells. Its defined roles include helping maintain retinal vascular barrier function [51], increasing expression of smooth muscle actin [52], and enhancing pericyte contraction [53], which have been suggested to help stabilize endothelial cells [54], [55]. We wished to evaluate the extent to which hASC-pericytes might also be influenced by TGF-β1 pre-treatment, if these treatment effects were analogous those observed with retinal pericytes, and if TGF-β1 might enhance hASC functionality within *in vitro* and *in vivo* assays of pericyte function and vascular stabilization. We first performed an hASC and bovine retinal endothelial cell (BREC) co-culture assay previously described for evaluating retinal pericytes [25]. Using this assay, we tested whether pre-conditioning the hASC and/or retinal pericytes with TGF-β1 would enhance these cells’ ability to inhibit BREC cell cycle entry in a contact-dependent manner. After 24–48 hours of pre-conditioning hASC or pericytes with TGF-β1, we found that BRECs contacting the TGF-β1 pre-treated hASCs are 14.4% less likely to be found in mitosis, as compared to co-cultures where endothelial cells contacted hASCs without TGF-β1 pre-conditioning (p<0.05). This result is comparable to the 15.7% decrease in endothelial S-phase entry in cells contacting pericytes (p<0.05) (Fig. 3A, B). BRECs in monoculture demonstrate a 23.1% decrease (p<0.01) in mitotic activity after direct constant exposure to TGF-β1 (Fig. 3A, B). Thus, pre-conditioned pericytes and hASCs contacting BRECs recapitulate most, but certainly not all, of the reduction in mitotic activity seen with direct TGF-β1 application to BRECs.

Furthermore, hASCs pre-treated with TGF-β1 demonstrated a 2.6 fold enhancement of contractility (n = 32 treated cells, 40 untreated cells, p = 0.0413) over untreated hASCs, as measured by the cells’ ability to deform an underlying silicone substratum (Fig. 3C, D). This is analogous to results obtained when comparing TGF-β1 and non-TGF-β1 treated endogenous bovine retinal pericytes [53]. TGF-β1 pre-treatment increased the fraction of hASCs highly expressing SMA by 15.3% without altering control β-actin levels (Fig. 3E, F), which may in part account for their enhanced contractility.

Having demonstrated *in vitro* effects of TGF-β1 pre-treatment on hASCs, we next sought to determine whether this would functionally improve their performance in protecting retinal vasculature from the effects of OIR. P6 NOD SCID pups were intravitreally injected with either 1,000 untreated or 1,000 TGF-β1 pre-treated hASCs in 0.5 µL PBS. When harvested at P12 after hyperoxia, eyes injected with TGF-β1 pre-treated hASCs demonstrated an 11.0% reduction in avascular area (n = 22, p = 0.01), compared to the contralateral eyes injected with untreated hASCs (Fig. 3G–I). This result suggests that TGF-β1 pre-treatment enhances hASC stabilization of retinal vessels *in vivo*.

The mechanism for TGF-β1 functional improvement of vessel stabilization remains unclear. The most straightforward explanation is that TGF-β1 pre-treatment enhances the pericyte functionality of our hASC-pericytes in a manner analogous to its effects on endogenous retinal pericytes. An alternative possibility, but one less directly supported by these results, is that our heterogeneous hASCs contain a subpopulation of pericyte precursor cells, which can be selected or functionally conditioned for endothelial protection and microvascular stabilization with TGF-β1.

### ASCs Prevent Retinal Capillary Dropout in the Akimba Model of DR

Having established that a functional retinal pericyte phenotype could be derived from hASCs, we next sought to examine their potential application for treatment of diabetic retinopathy. The recently published Akimba model features large-scale retinal capillary dropout, vessel constriction, edema, fibrosis, and bleeding in an immunocompetent diabetic mouse [35]. These findings demonstrate that this animal model replicates many more of the characteristics of severe human DR than other available models. Briefly, diabetic Ins2^Akita^ mice were crossed with Kimba mice that express human VEGF165 under control of the rhodopsin promoter. The resultant Akimba pups demonstrated characteristic early onset hyperglycemia, followed by substantial weight loss and retinopathy, exhibiting profound retinal capillary dropout by 2 months of age. Because Akimba mice are fully immunocompetent, we used allogeneic murine adipose-derived stem cells (mASCs) derived from the same background strain.

**Figure 4:**
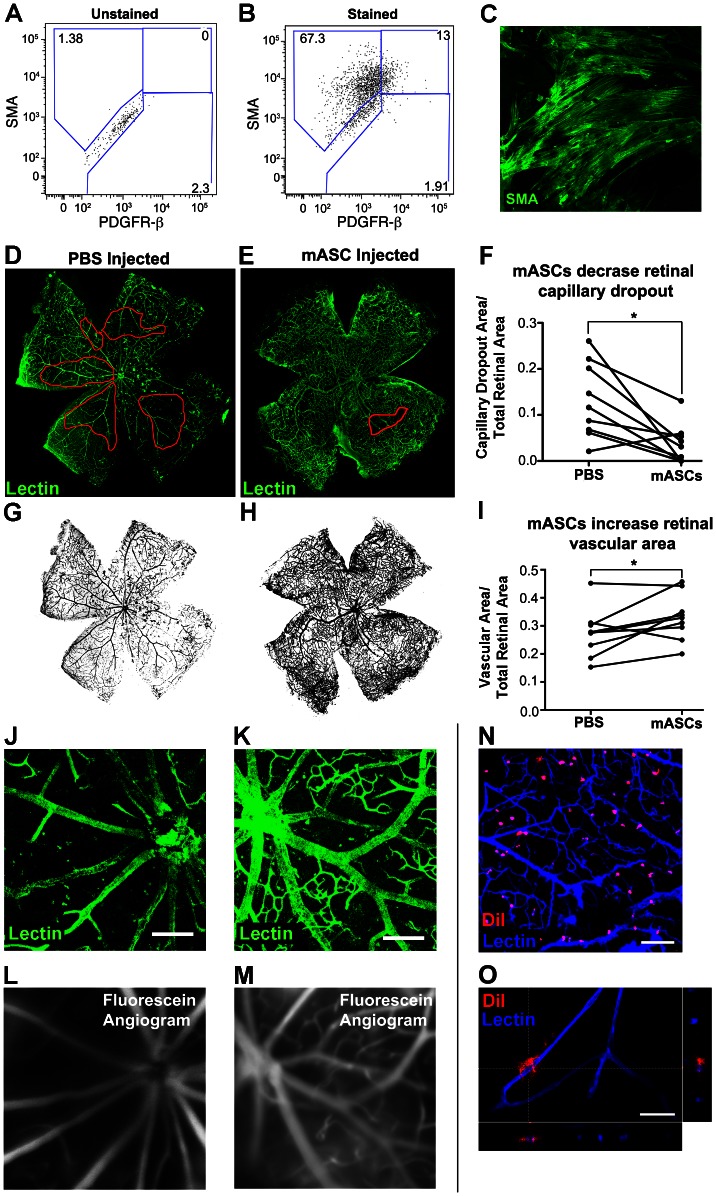
TGF-β1 treated mASCs prevent long-term retinal capillary dropout in diabetic retinopathic Akimba mice. **A**, **B**, Passage 4 mouse adipose-derived stem cells (mASCs) were found to express SMA (80.3%), PDGFR-β (14.9%), or both (13%) compared to unstained controls. **C**, mASCs also express SMA in a typical pattern *in vitro*, similar to hASCs. **D–M**, TGF-β1 treated mASCs were injected at P9 in Akimba mice, with PBS carrier control injected in the contralateral eye. Compared to eyes injected with PBS carrier control (**D**), circumscribed area of retinal capillary loss in 8 week old Akimba mice was substantially decreased in eyes injected with mASCs (**E**), as indicated by a 79% reduction of capillary dropout area (**F**, n = 9, p = 0.01). Similarly, PBS-injected eyes (**G**) demonstrated an 18% lower vascular area/total retinal area ratio than contralateral mASC-injected eyes (**H**), (**I**, n = 9, p = 0.05). Preservation of retinal capillaries is revealed at higher magnification by both lectin-stained retinal whole mounts (**J**, **K**) and *in vivo* fluorescein angiography (**L**, **M**). Note that images **J–M** were taken from the same mouse. **N**, After injection with DiI labeled mASCs at 5 weeks of age, subsequent harvest at 9 weeks revealed substantial integration of mASCs into an Akimba retina. **O**, Di-I labeled mASC wrapping around Akimba retinal microvessel. XZ and YZ planes are also displayed. Scale bars: **J**, **K = **150 µm, **N = **100 µm, **O** = 25 µm.

We first verified that mASCs, isolated from the epididymal fat pad and cultured in the same conditions as hASCs, displayed characteristic pericyte markers. As expected, mASCs were found to express abundant steady state protein levels characteristic of native microvascular pericytes, including SMA and PDGFR-β as measured by both flow cytometry and immunohistochemistry (Fig. 4A–C). That 14.9% of mASCs were positively stained with PDGFR-β is consistent with similar findings in rat [56]. When injected at 5 weeks of age and harvested at 9 weeks of age, we found mASCs readily integrate and associate with Akimba retinal microvasculature (Fig. 4N, O).

We next tested the hypothesis that mASCs can prevent diabetic retinal microvascular dropout by injecting DiI-labeled, TGF-β1 pre-conditioned, mASCs into the vitreous of P9 Akimba pup eyes. As before, PBS was injected as a carrier control in the contralateral eye. Two months after injection, and subsequent verification that each mouse was diabetic, retinae were harvested and retinal microvessels stained with lectin as before. Over the course of two months, no teratoma formation was observed in any of the 18 dissected eyes. Wholemount analysis of retinal vessels revealed a 79% reduction in the area of capillary dropout in retinas treated with mASCs compared to contralateral PBS injected controls (n = 9, p = 0.012) (Fig. 4D–F). Complimentary analysis of total vessel area showed a 16% reduction in absolute loss of retinal vasculature in mASC-injected compared to contralateral PBS control-injected eyes (n = 9, p = 0.047– paired t-test) (Fig. 4G–I). Despite this dramatic reduction in vascular dropout, we interestingly found no significant reduction in the number of microaneurysms in mASC injected compared to PBS injected eyes (n = 9, p = 0.43– paired t-test). However, given the low frequency of microaneurysms in both cell injected and control eyes, greater numbers of animals may be needed to demonstrate a significant difference.

Prior to retinal harvest, mice were also imaged *in vivo* using fluorescein angiography with customized Cantor Nissel contact lenses and a Heidelberg Spectralis Retinal Imager (Fig. 4L, M). Importantly, we found that protected retinal microvessels in the mASC-injected eyes remain functional, as assessed by comparing the microvasculature by topical wholemount lectin staining (Fig. 4J, K) with the corresponding fluorescein angiography (Fig. 4L, M). Taken together, these results suggest that mASCs display a pericyte phenotype analogous to hASCs and show a similar capacity to protect retinal microvessels functionally from loss in the setting of diabetic retinopathy.

### Conclusions

We have demonstrated the ability of ASCs to differentiate into pericytes, as determined by pericyte-specific marker expression, *in vitro* confirmation of perciyte function, and response to TGF-β1 treatment that is analogous to that of endogenous retinal pericytes. When injected intravitreally, ASCs enhance retinal microvascular stabilization in three independent pre-clinical murine models of retinopathic vasculopathy. Specifically, ASC-derived pericytes: 1. When injected prior to OIR, protect the retinal vasculature against central vascular dropout, 2. When injected post-OIR insult, accelerate vascular regrowth and recovery of the central retinal vasculature [36], and 3. When injected at early postnatal time points, protect against capillary dropout in the Akimba murine model of DR [35]. *Ex vivo* treatment of ASC-derived pericyte progenitors with TGF-β1 recapitulates the response of endogenous retinal pericytes to TGF-β1, with enhanced pericyte marker expression, improved performance in *in vitro* functional assays and enhanced *in vivo* protection of the retinal microvasculature.

There has been increasing recognition of the role that dysregulation between pericytes and endothelial cells plays in the susceptibility of the retinal vasculature to aberrant angiogenesis and vasculopathies such as DR, age-related macular degeneration, and ROP [7], [52]. DR, a disease that proceeds over many years from initial pericyte dysfunction to microvascular degeneration, may be particularly well suited for cell-based therapies. However, in the absence of a readily available supply of human retinal pericytes for transplantation, identifying a plentiful, surgically accessible, and potentially autologous alternative could offer great therapeutic potential.

Our findings demonstrate that ASCs can differentiate into pericytes and, when exogenously injected, will incorporate within the retinal microcirculation with maintained pericyte marker expression, recapitulating results using these cells in other non-ocular model systems [37]. Our studies also help establish that injection of an exogenous pericyte progenitor population can functionally protect retinal microvessels against profound capillary dropout in a novel pre-clinical model of retinal vasculopathy. Given that ASCs are perivascularly located and thought to function in adipose vascular support [57], it is perhaps not surprising that they would be able to incorporate and affect the retinal vasculature in similar manner, though our studies are importantly the first direct demonstration of this.

Further work is clearly needed to illuminate the dynamic control of retinal vessel formation and degeneration in retinal vasculopathies, such as DR and ROP, especially as it pertains to the observed functional effects of injected ASCs. Exogenous insulin-like growth factor-1, along with other paracrine signals, has been shown to modify retinal vascular stabilization in OIR [58]. ASCs readily secrete insulin-like growth factor-1, more so than other types of human mesenchymal stem cells [59], and might be one method by which ASCs exert their influence on retinal vessels. Functional stabilization of retinal vasculature by ASCs, even in areas of the retina where no direct incorporation of ASC progenitors on retinal vessels is observed, lends support to the idea that ASCs exert their effects, at least in part, by conditioning the retinal microenvironment. If verified, this mechanism of action would bode well for future translational applications. It would suggest that encapsulated cells injected into the vitreous might be sufficient to stabilize retinal vessels, without requiring direct vascular integration [42], [60].

Additionally and importantly, recent work from our group has revealed that pericyte dysfunction, rather than pericyte loss, may be ‘rate-limiting’ in determining whether retinal endothelial growth and microvascular proliferative disorders ensue as outcomes or complications accompanying conditions such as diabetes [25], [28]. In light of these findings, we intend to explore further the extent to which injected ASCs may help maintain normal function of the native pericyte population in the face of toxic environments, such as chronic hyperglycemia. Given ASC’s ability to survive the relatively greater hypoxic conditions of adipose tissue as compared to the eye, they may be particularly well suited to respond to such insults.

Despite the remaining work required to translate ASC stem cell therapy to the clinic, our results suggest that stem cell based strategies for retinal vasculopathies may one day allow a shift in focus from late destructive laser treatment of hypoxic retina to earlier preventative interventions aimed at stabilizing existing retinal microvasculature. Our present findings suggest this may be accomplished using ASCs through both direct contact with retinal microvessels, as well as more general paracrine conditioning of the retinal microenvironment, both of which can prevent vessel loss and retinal hypoxia from occurring in the first place. The consistent and robust microvascular stabilizing properties of ASC-derived pericytes offer hope that such a regenerative treatment for retinal vasculopathies, including diabetic retinopathy, may be attainable.

## Supporting Information

Figure S1 **hBMSCs fail to accelerate hypoxic revascularization.** Compared to contralateral PBS injected controls (**A**), eyes injected with unsorted hBMSCs (**B**) at P12 and harvested at P14 showed no statistical difference in rate of revascularization of the central retina at P14 (**C**). However, when compared to PBS contralateral controls (**D**), eyes injected with hASCs again demonstrated a statistically significant decrease in central retinal capillary dropout (**F**, 16.4% reduction, p = 0.03, n = 5).
(TIF)

Figure S2 **P2 hASC injections do not impact normal retinal vascularizaition.** Compared to contralateral PBS injected controls (**A**), eyes injected with hASCs (**B**) at P2 and harvested at P7 (without exposure to hyperoxia) displayed no obvious differences in vascular coverage, density, or morphology.
(TIF)
