# Correction: Pericytes Derived from Adipose-Derived Stem Cells Protect against Retinal Vasculopathy

**DOI:** 10.1371/annotation/679017bf-abd5-44ce-9e20-5e7af1cd3468

**Published:** 2013-09-17

**Authors:** Thomas A. Mendel, Erin B. D. Clabough, David S. Kao, Tatiana N. Demidova-Rice, Jennifer T. Durham, Brendan C. Zotter, Scott A. Seaman, Stephen M. Cronk, Elizabeth P. Rakoczy, Adam J. Katz, Ira M. Herman, Shayn M. Peirce, Paul A. Yates

An NIH grant to the last three authors (IMH, SMP and PAY) was incorrectly omitted from the Funding Statement. The number of the grant is: EY022063-01. 

