# Supplementary figures and images for: Pericytes Derived from Adipose-Derived Stem Cells Protect against Retinal Vasculopathy
Source: PLoS One. 2013 May 31;8(5):e65691. doi: 10.1371/journal.pone.0065691 (PMC3669216; doi:10.1371/journal.pone.0065691)

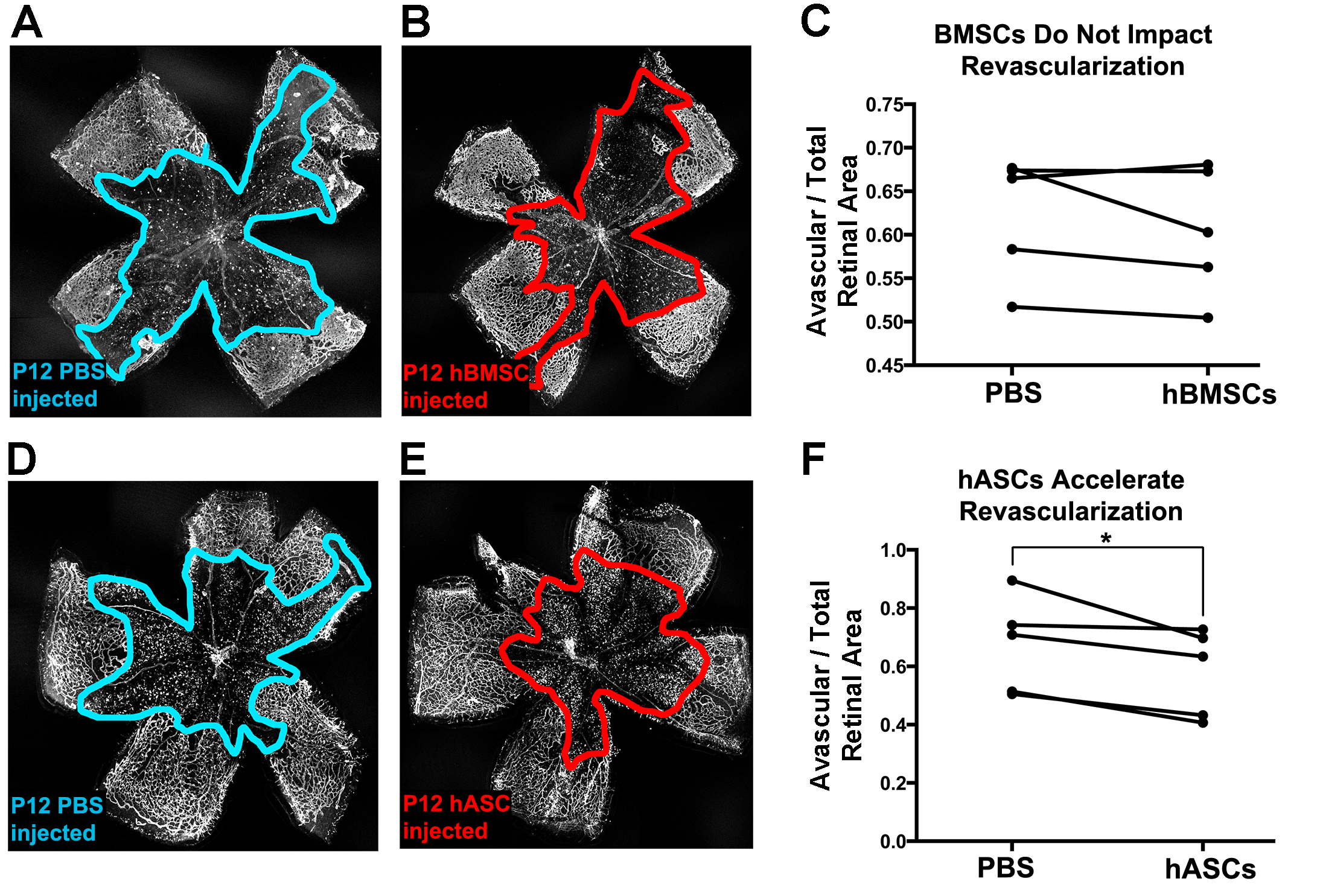

Supplement: Figure S1 — hBMSCs fail to accelerate hypoxic revascularization. Compared to contralateral PBS injected controls (A), eyes injected with unsorted hBMSCs (B) at P12 and harvested at P14 showed no statistical difference in rate of revascularization of the central retina at P14 (C). However, when compared to PBS contralateral controls (D), eyes injected with hASCs again demonstrated a statistically significant decrease in central retinal capillary dropout (F, 16.4% reduction, p = 0.03, n = 5). (TIF) [file pone.0065691.s001.tif]

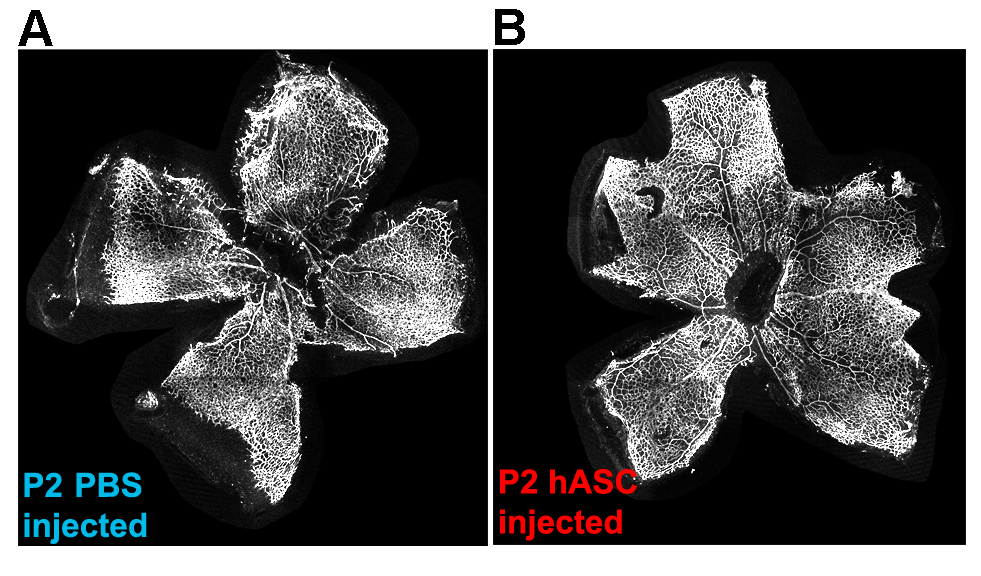

Supplement: Figure S2 — P2 hASC injections do not impact normal retinal vascularizaition. Compared to contralateral PBS injected controls (A), eyes injected with hASCs (B) at P2 and harvested at P7 (without exposure to hyperoxia) displayed no obvious differences in vascular coverage, density, or morphology. (TIF) [file pone.0065691.s002.tif]
